# Ecofriendly Water-Soluble Binders for Precision Ceramic Moulds in Aerospace Turbine Casting: Process Development and Performance Evaluation

**DOI:** 10.3390/ma18102329

**Published:** 2025-05-16

**Authors:** Marcin Małek, Marcin Wachowski, Janusz Kluczyński

**Affiliations:** 1 Faculty of Civil Engineering and Geodesy, Military University of Technology, 2 Gen. S. Kaliskiego St., 00-908 Warsaw, Poland; 2 Institute of Robots & Machine Design, Faculty of Mechanical Engineering, Military University of Technology, 2 Gen. S. Kaliskiego St., 00-908 Warsaw, Poland; marcin.wachowski@wat.edu.pl (M.W.); janusz.kluczynski@wat.edu.pl (J.K.)

**Keywords:** ceramic-casting moulds, aircraft turbine, binders, nickel superalloys

## Abstract

The paper presents the development of an ecofriendly ceramic moulding system for the precision casting of aircraft turbine components from nickel superalloys using water-soluble binders. The motivation was to eliminate hydrolysed ethyl silicate (HES) due to its environmental and occupational hazards. Two water-based binders (K + M)—Keysol (for the primary layer) and Matrixsol (for the backup layers)—were evaluated against the standard HES-based system. A comprehensive comparative analysis was conducted including microstructure, phase composition, wettability, mechanical, thermal, and gas permeability properties. The developed K + M ceramic moulds achieved a bending strength of 12.4 MPa after annealing, average surface roughness (Ra) below 5 µm, and open porosity of 29.1%, indicating excellent strength and permeability. Thermal conductivity increased from 0.3 W/mK to 2.0 W/mK between 22 °C and 1400 °C. The wetting angle of water-based binders was higher (Keysol: ~36°) compared to HES (~5°), resulting in more stable surface morphology. Gas permeability was maintained at 5.6 × 10^−9^ cm^2^ at 1100 °C, ensuring effective degassing during casting. The results demonstrate that the K + M system can replace HES in production while improving safety and reducing environmental impact, making it suitable for industrial-scale implementation in the aerospace sector.

## 1. Introduction

The requirements regarding the functional properties of a ceramic mould depend on many factors. The basic ones are the type of alloy and the type of process and pouring method for which the mould is intended. An important factor is the strength of the mould in the raw state and after annealing. Additionally, the ceramic mould should be characterised by a high resistance to thermal shocks and good chemical stability—no reaction with the liquid alloy. The mould material should also have adequate gas permeability and thermal conductivity [1,2,3,4]. These properties determine the displacement of air through the mould walls by the flowing liquid metal. Air should quickly penetrate the network of open pores outside the mould, thus preventing the formation of internal defects and microporosity in the castings. Additionally, the mould material should have a low linear thermal expansion coefficient in the temperature range of 600–1500 °C [5,6,7,8,9]. Depending on the requirements and application, the ceramic-casting mould is made up of several or a dozen ceramic layers that differ in physical and thermal properties. The first layer is the basic one—prime coat. It is in direct contact with the liquid alloy; therefore, the material of this layer should be characterised primarily by chemical inertness towards the liquid alloy and its alloy additives. In addition, it ensures surface reproduction; therefore, when applied, the sand that forms this layer must have the appropriate viscosity [10,11,12,13]. Subsequent construction layers (backup) ensure specific mechanical properties of the form. They cannot be damaged during the pouring process and should be flexible at the same time. Then, during the casting crystallisation process, no large stresses are generated, which often lead to the formation of casting defects [3,11,12,13,14,15].

The materials used to build the ceramic form are classified into several groups, including binders, ceramic powders, loose powders, and auxiliary materials and antifoaming and wetting agents. The binder is the basic and most important component of the moulding sand. It acts as a connector connecting the remaining components of the sand. Therefore, it is characterised by specific physical and chemical properties that ensure good heat resistance, chemical stability of the mould, and high smoothness of its internal surface [12]. The drying process involves shrinkage of the mould material and chemical reactions of the binder and components of the moulding sand. Often, this results in the formation of defects in ceramic moulds when poured with liquid metal. Therefore, the binder should ensure good strength properties between individual layers. The binder used in the production of ceramic-casting moulds is characterised primarily by the following: good rheological properties, good resistance to sedimentation, good stability of the chemical composition, high chemical inertness towards the liquid alloy, and good miscibility with the remaining components of the ceramic mass [15]. Ceramic powders form the matrix of the ceramic mixture. Their appropriate selection significantly influences the physical and chemical properties of the form. The required properties of powders are mainly resistance to high temperature, low thermal expansion, and lack of polymorphic transformations [16,17]. The most commonly used ceramic powders for casting mould production are aluminium, zirconium, and yttrium oxides. Currently, scientists are conducting research on the use of new compounds based on silicon carbide, which, thanks to its high thermal conductivity, would reduce the production time of the finished casting by removing heat from the inside of the mould faster. This would also allow for increasing the mechanical properties of the manufactured aircraft turbine components by fragmenting the microstructure and reducing the number of mould cracks due to the very high resistance of silicon carbide to thermal shocks. These works have been the subject of numerous patent applications and publications [12,13,14,15,16]. Auxiliary materials include antifoaming agents, wetting agents, bactericidal and fungicidal agents, and pH stabilisers. They are used to ensure good coverage of the wax model by the ceramic moulding mass, to increase its shelf life, and to reduce the tendency for air bubbles to form during mixing. A large number of bubbles increases the porosity of the mould. Hence, its mechanical properties decrease, also during the rapid removal of heat from the liquid metal—excessive shrinkage of the casting [17,18,19,20].

After mixing, the materials presented form the moulding sand used in the production of moulds. The physical and chemical properties of the moulding sand must ensure good coverage of the wax model and provide the mould with appropriate functional properties. To produce ceramic moulds intended for precision casing of aircraft engine turbine components, the domestic industry uses binders mostly based on alcohol compounds, including hydrolysed ethyl silicate (HES). It has a density close to 0.887 g/cm^3^ of water. Among competitive ecological materials, binders using colloidal silica (CS), a hydrosol of silicic acid in the form of an aqueous suspension, have the highest consumption in precision casting. The agent that binds the binder to the ceramic powder particles is silica gel, which is formed by evaporating water from the sol. Colloidal silica, compared to binders based on alcohol compounds, is neutral to the natural environment and harmless to workers [21,22,23,24,25,26,27]. Hydrolysed ethyl silicate (HES), despite its widespread use in ceramic mould manufacturing, presents significant occupational and environmental hazards. As an alcohol-based compound, HES releases volatile organic compounds (VOCs) during application and curing, contributing to air pollution and posing direct health risks to foundry workers, including skin and respiratory tract irritation, headaches, and long-term organ toxicity. Furthermore, the flammability of alcohol-based systems increases the risk of workplace accidents. In recent years, increasingly stringent environmental regulations, such as the European Union’s REACH framework and the growing adoption of ISO 14001-compliant environmental management systems in industry, have driven the search for sustainable alternatives. The shift toward water-soluble binder systems responds not only to regulatory compliance but also to broader trends in green manufacturing and corporate responsibility. Water-based binders such as colloidal silica eliminate the need for organic solvents, significantly reduce toxic emissions, and offer improved worker safety without compromising material performance. These considerations underline the urgency and industrial relevance of developing an effective ecological substitute for HES in the precision casting of superalloy components. 

The aim of this work was to develop an ecological process for producing ceramic-casting moulds for the precise casting of low-pressure aircraft engine blades from nickel superalloys using water-soluble binders. The paper also presents the results of research on the development of an ecological binder and replacement of the binder based on hydrolysed ethyl silicate (HES). During the research work on replacing the HES binder, because of the lack of data on the properties of the hydrolysed ethyl silicate-based system, an attempt was made to independently determine all the physical and chemical properties of the binder, powders, additives, and moulds in the conditions of a precision foundry. The definition of the correct physical, chemical, and mechanical properties of the developed moulding system was based on the full characteristics of the moulding system currently used in production conditions, manufactured using hydrolysed ethyl silicate (HES). The key innovation of this work lies in the complete technological replacement of the alcohol-based hydrolysed ethyl silicate (HES) binder system with a water-soluble, ecofriendly binder system (Keysol and Matrixsol) in the production of multilayer ceramic moulds for the precision casting of nickel-based superalloy turbine components. Unlike previous studies that investigated water-based systems at the level of individual layers or in simplified laboratory trials, our approach involved the following:

1. Comprehensive characterisation of the new binder system, including particle size distribution of silica nanoparticles, zeta potential, viscosity behavior, solid-phase content, and thermal stability using advanced techniques (e.g., SEM, TGA, FTIR, DSC, dilatometry, and porosimetry);
2. Multistage experimental validation of mould properties—mechanical (bending strength, Young’s modulus, and Weibull modulus), surface (roughness and wetting angle), microstructural (XRD and SEM), thermal (conductivity, specific heat, and expansion), and functional (porosity and gas permeability)—at each stage of the casting process (raw, fired, and annealed);
3. Head-to-head benchmarking of the new system against a conventional industrial standard (HES-based), demonstrating superior or comparable performance, with statistical validation of key improvements (e.g., post-annealing bending strength increased by 9.5%, surface roughness reduced by ~18%, and VOC emissions nearly eliminated);
4. First reported implementation of the Keysol/Matrixsol system in a full seven-layer mould architecture for aerospace-grade turbine blades, including pilot-scale trials under production-mimicking conditions;
5. Environmental and safety impact analysis, emphasizing the real-world applicability of the findings, which is rarely addressed in academic studies focused on binder formulation alone.

## 2. Materials and Research Methodology

### Research Materials

To create an innovative ceramic mould, it was necessary to test and select the optimal properties of the ceramic powders, binders, and moulding sand resulting from mixing the base materials. Then, after the appropriate tarting components were selected, the ceramic moulds were obtained and examined by comparing their properties with those of previously used ceramic moulds. Chemical composition of ceramic mixtures for individual layers of the casting mould are presented in Table 1. When optimising base materials, several materials were taken into account, such as the following:

- ceramic powders: aluminium oxide 200# and 325#, Molochit 120;
- binders: Keysol, intended for the first layer of the ceramic mould, and Matrixsol.

Appropriately selected ceramic powder and binder were used to produce the sand, which was intended for the first layer and the structural layers of the ceramic mould. The final stage was the creation of optimised ceramic forms and comparison of them with the currently used solutions. Therefore, to select appropriate powders, binders, and moulding sands to produce innovative ceramic forms, a number of studies were carried out.

A Hitachi S-3500L scanning electron microscope (Ibaraki Prefecture, Japan) was used to observe the microstructure of powders, binders, mould sand, and ceramic mould. A beam of backscattered electrons (BSE) and an accelerating voltage of 5–20 kV were used. The powder particles were placed for observation on a carbon tape. The surface morphology of the binders after drying and applying to the carbon tape was examined using a high-resolution Joel-Jem 3010 scanning electron microscope (Akishima, Tokyo, Japan) and a Hitachi S5500 scanning electron microscope (Ibaraki Prefecture, Japan). The accelerating voltage used for the JEOL JEM 3010 microscope was 300 kV and for the Hitachi S5500 was 30 kV. The SEM images obtained constituted the basis for quantitative analysis of the microstructure using the Micrometer programme v1.0 (Warsaw University of Technology, Poland).

Powder particle size measurements were performed using a Horiba LA 950 laser particle size analyser (Tokyo, Japan), using the low-angle laser light scattering (LALLS) method.

Measurements of the chemical and phase composition of powders, binders, sand moulding, and ceramic mould were made using a WDXRF Bruker S4 Explorer X-ray fluorescence spectrometer (Billerica, MA, USA) and a Bruker D8 Advance powder diffractometer (Billerica, MA, USA).

Zeta potential measurements were performed for ceramic powders to assess the electrokinetic stability as a function of pH. The tests were performed using the Zetasizer Nano ZS analyser (Malvern, UK).

Solid-phase content tests were carried out for binders to properly determine the specific solid-phase content, and thus the appropriate formula of pouring moulding sand. The solid-phase particle content was determined by determining the dry weight of the sample. The mass of the binder before and after centrifugation was determined. The remaining mass corresponded to the solid-phase content.

For the binders, the wetting angle was tested on PE foil using the Wetting angle System OCA goniometer. The wetting angle value was given as the average of 10 measurements. The wetting angle test was also carried out for ceramic moulds using the lying drop method and joint heating of the alloy and mould to the experimental temperature. Then, the alloy and mould were heated at this temperature and cooled. The alloy and the mould are in constant contact during the test. During the test, images of a drop of superalloy (IN713C) placed on a flat ceramic mould substrate were recorded. These images were the basis for determining the value of the wetting angle Θ, also for the geometric measurements of the drops. The experiment was carried out in an argon atmosphere with a constant pressure of 1.3·10^−2^ Pa. Immediately before being placed in the testing chamber, the samples were cleaned with isopropyl alcohol.

The chemical composition of the binders was analysed using the Fourier transform infrared (FTIR) method. The compounds were analysed in the wave number range of 4000–400 cm^−1^ and the resolution in the range of 0.5–32 cm^−1^.

Thermal analysis of binders using the thermogravimetry technique was performed using a TA Instruments DCSQ1000 calorimeter (New Castle, DE, USA). The tests were carried out under a nitrogen atmosphere using aluminium crucibles. Samples weighing 10 mg were heated and cooled at a constant rate of 20 °C/min in the temperature range of 20–1000 °C.

To test the relative viscosity, that is, the mass flow time, a Elcometer Zahn 4 draw cup (Edge Lane, UK) with a nizzle diameter of φ = 4.3 mm was used. The flow time was examined using a digital stopwatch from the moment of fully filling the cup through its immersion in the pouring mass and determining the free flow of the slurry until the flow was interrupted.

Dynamic viscosity measurements were carried out on the Anton Paar MC 102 rheometer (Graz, Austria) using the coaxial cylinder method in the shear rate ranges of 1.3–200 and 200–1.3 1/s.

The mechanical properties of the moulds were determined in a three-point bending test of prepared ‘beam’ samples made of ceramic mould material. The tests were carried out according to the ASTM D790 standard. The adopted support spacing was 25 mm. Using an electromechanical extensometer for a measurement length of 25 mm, the beam deflection arrow was determined. Weibull’s statistical theory was used to determine the bending strength.

The surface roughness of the mould was determined using an optical profilometer NT9300 (Plainview, NY, USA) with a magnification of 11.5, 28.4, and 101.5× on “beam” samples cut from a ceramic mould. The values of the Ra surface parameter were determined.

The thermal properties of the ceramic moulds were tested using a DIL 402/4/1G Netzsch dilatometer (Selb, Germany). The following were determined: the temperature of phase transformations in the solid state, changes in the length of the samples as a function of temperature and the density of their materials, and the values of the linear thermal expansion coefficients as a function of temperature. Specific heat was determined using a DSC 404 C Pegasus differential scanning calorimeter from Netzsch (Selb, Germany) according to the ASTME 1269 standard [28]. The thermal conductivity coefficient was determined using the Netzsch LFA 427/4/G flash laser device (Selb, Germany).

The porosity of the tested ceramic forms was determined using mercury porosimetry (Auto Pore II 9220 porosimeter, Micrometrics, Selb, Germany). The sample was placed in a penetrometer with a constant of 21.63 µL/pF, a mass of 67.9740 g, and a capillary volume of 6.6766 mL. The penetrating liquid was mercury with a density of 13.5389 g/cm^3^ and a surface tension of 0.485 J/m^2^. The capillary wetting angle with liquid mercury was Θ = 130°. The samples were weighed before measurement and placed on the penetrometer. Redetermining the mass of the penetrometer after filling with mercury allowed determining the apparent density of the tested samples of ceramic materials.

Ceramic mould gassing was determined by continuously measuring the volume of the residual gases using a gas spectrometer while heating them in a vacuum (1500 °C). Throughout the test cycle, measurements of the pressure in the test chamber and the composition of the residual gases were made. Temperature values were measured on the research table, next to the table, above the table, and on the radiator.

The gas permeability of the forms was determined on the basis of the constructed measurement station and the Darcy relationship described by Equation (1) [1,2]. To determine the gas permeability, air was passed through a spherical sample of the ceramic mould, and the pressure and air flow values flowing through the sample were recorded.

(1) $$K=\frac{MQ\prime D}{PA}$$

where the following variables are used:

K—permeability [cm^2^];

M—dynamic viscosity of air at a given temperature [(kg/m*s)*10^−5^];

D—thickness of the mould wall (mould diameter—ping-pong ball diameter) [mm];

A—mould surface area [mm^2^];

P—air pressure [mmH_2_O];

Q′—air flow value at measurement temperature [cm^3^/min].

The dynamic viscosity of air (M) in a certain temperature range of 30–1300 °C is directly proportional to the temperature. For a certain temperature range, this function takes the form of a linear relationship. This allows a direct reading of the M value for a specific temperature of the measurement. The air flow value (Q′) depends on the ambient temperature. The measurement is carried out in centres with different temperatures (laboratory furnace), so the working medium, which is air, also has different temperatures. Taking this fact into account, a correction should be made for the air flow value at the measurement temperature described by Equation (2) [1,2].

(2) $$Q\prime =\frac{QT\prime }{T}$$

where the following variables are used:

Q′—air flow value at measurement temperature [cm^3^/min];

Q—air flow value at room temperature [cm^3^/min];

T′—value of measurement temperature [K];

T—room temperature value [K].

The research material were spherical ceramic forms produced by immersion. All samples consisted of 7 layers, with the last layer playing only a sealing role. It was produced by immersing the sample in a ceramic mass, without sprinkling it. The samples were allowed to dry freely at room temperature. After drying, the samples were fired at a temperature of 760 °C. The furnace was heated and cooled together with the samples to avoid cracking of the material.

**Figure 1:**
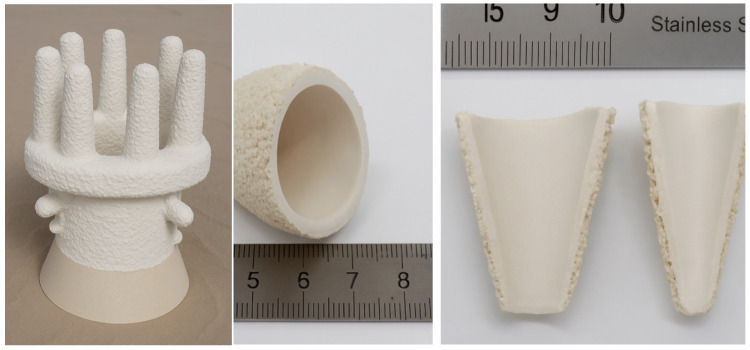
Structure of the ceramic mould.

Thermal and weight methods were used to determine the kinetics of the drying process of the mould layer. In the case of the thermal method, three thermocouples embedded in the wax model were used. Then, the blade model was the basis for producing a ceramic mould according to the developed moulding system. After each layer was applied, the thermocouples were connected to the Hioki 8875 recorder (Nagano, Japan). Temperature values were measured over a period of 10 s. The values obtained for the mould temperature close to the ambient temperature were interpreted as the time for the water to evaporate from the layer and the end of the drying process. Ceramic moulds were produced using the weight method on a wax model according to the developed moulding system. After applying each layer, the change in model mass over time was determined in a period of 10 s. Reaching a plateau during measurement was interpreted as the evaporation of water and the drying of the mould layer. This method also makes it possible to determine the mass of evaporated water in the individual layers of the ceramic mould. Figure 1 shows the experimental workflow and the multilayer structure of the ceramic mould, indicating the composition and function of each layer.

Keysol and Matrixsol (K + M) are water-soluble, two-component binder systems designed for application in ceramic mould production. Keysol consists of an acrylic-vinyl copolymer concentrate, comprising approximately 20–40 wt% of the formulation, and an aqueous dispersion of amorphous silica nanoparticles with an average particle diameter ranging from 12 to 18 nm. The polymer phase facilitates the formation of an initial organic matrix, enhancing the mechanical cohesion of the uncured ceramic layer. Matrixsol, applied to the backup structural layers, incorporates 2,2′,2″-(hexahydro-1,3,5-triazin-1,3,5-triyl)triethanol as a crosslinking and stabilizing component (70–100 wt% active substance) combined with a colloidal silica phase, where the SiO_2_ content is approximately 33.7 wt%.

Although both binders were obtained from commercial sources (Ransom & Randolph, Maumee, OH, USA), their physical and chemical properties were fully characterised within the study to ensure compatibility with ceramic powders and to optimize process parameters. The morphology and dispersion of the silica nanoparticles were confirmed using scanning electron microscopy (SEM) and laser diffraction particle sizing (DLS), while zeta potential measurements confirmed high electrokinetic stability over a broad pH range, preventing unwanted agglomeration. Thermal stability was assessed via thermogravimetric analysis (TGA), and functional groups present in the binders were identified using Fourier-transform infrared spectroscopy (FTIR).

At the microscale, the interaction between the binder and ceramic particles such as aluminium oxide (Al_2_O_3_) and Molochite is governed by both physical adsorption and chemical bonding. Initially, the silica nanoparticles adsorb onto the surface of the ceramic grains through hydrogen bonding and van der Waals interactions. Upon drying, water evaporation induces a sol-gel transition in which silanol groups (Si–OH) condense to form Si–O–Si bridges, resulting in a rigid three-dimensional network that acts as an inorganic binder. The organic polymer in Keysol ensures uniform wetting and suspension stability before gelation, while the alkaline nature of Matrixsol (pH ~10.3) promotes enhanced surface interaction with aluminosilicates and better penetration into porous ceramic matrices. This dual mechanism—organic polymer film formation and inorganic silica gel bonding—provides excellent interlayer adhesion, dimensional stability, and mechanical strength of the final ceramic mould. The integration of polymer–nanoparticle synergy ensures reproducibility, ease of processing, and compatibility with multilayer mould designs required in aerospace precision casting.

## 3. Research Results and Discussion

### 3.1. Moulding Powders

**Figure 2:**
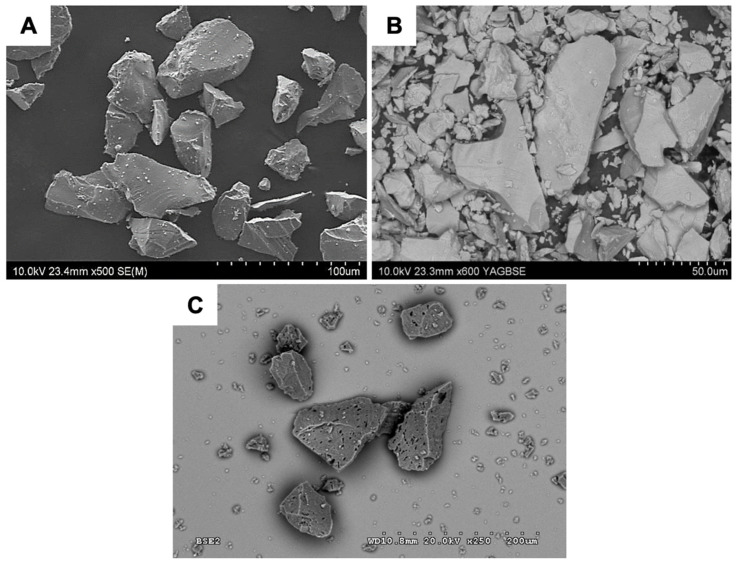
SEM images of powder particles: (**A**) Al_2_O_3_ 200#, (**B**) Al_2_O_3_ 325#, and (**C**) Molochite 120.

**Figure 3:**
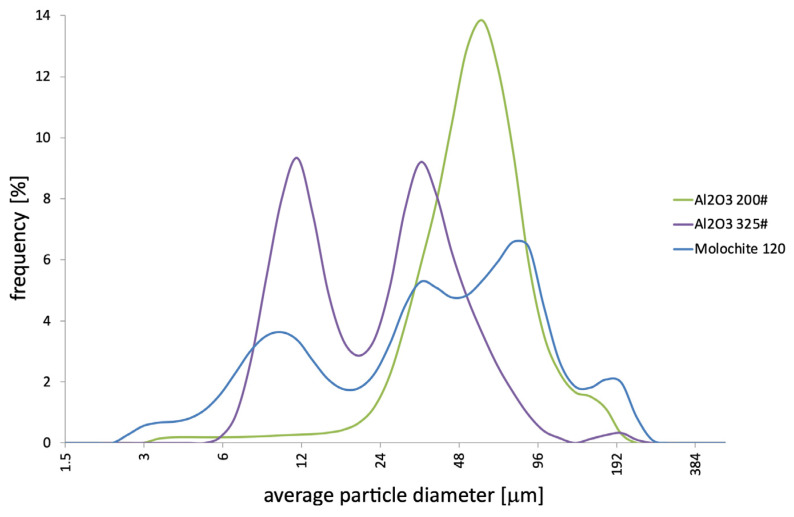
Distribution of the average particle diameter of powders used for the first layers: Al_2_O_3_ 200#, Al_2_O_3_ 325#, and Molochite 120.

The analysed powders have the typical morphology of particles used in the production of ceramic moulds. They are characterised by an irregular shape, sharp edges, and a developed surface (Figure 2A–C). The distributions obtained for the average particle diameter of the powders used for the first layer are presented in Figure 3 and Table 2. The analysis was performed in an aqueous environment. It has been established that powders are characterised by the following characteristics:

- Al_2_O_3_ (200#)—The powder has a wide range of particle sizes from 3.4 to 200 µm. The average particle diameter is 57.2 µm; the distribution obtained is symmetrical and has a maximum, d = 58.95 µm.
- Al_2_O_3_ (325#)—The powder has a wide range of particle sizes from 5.8 to 229 µm. The average particle diameter is 27.6 µm; the obtained distribution is asymmetric and has two maximums, d = 11 µm and d = 34 µm, which can indicate the presence of agglomerates.
- Molochite 120—The powder has a wide range of particle sizes from 2.60 µm to 262.68 µm. The average diameter of the powder particles is 51.22 µm; the distribution obtained is asymmetric and has three maximums, including d = 10.10 µ, d = 39.23 µm, and d = 88.58 µm, which may indicate the presence of agglomerates.

**Figure 4:**
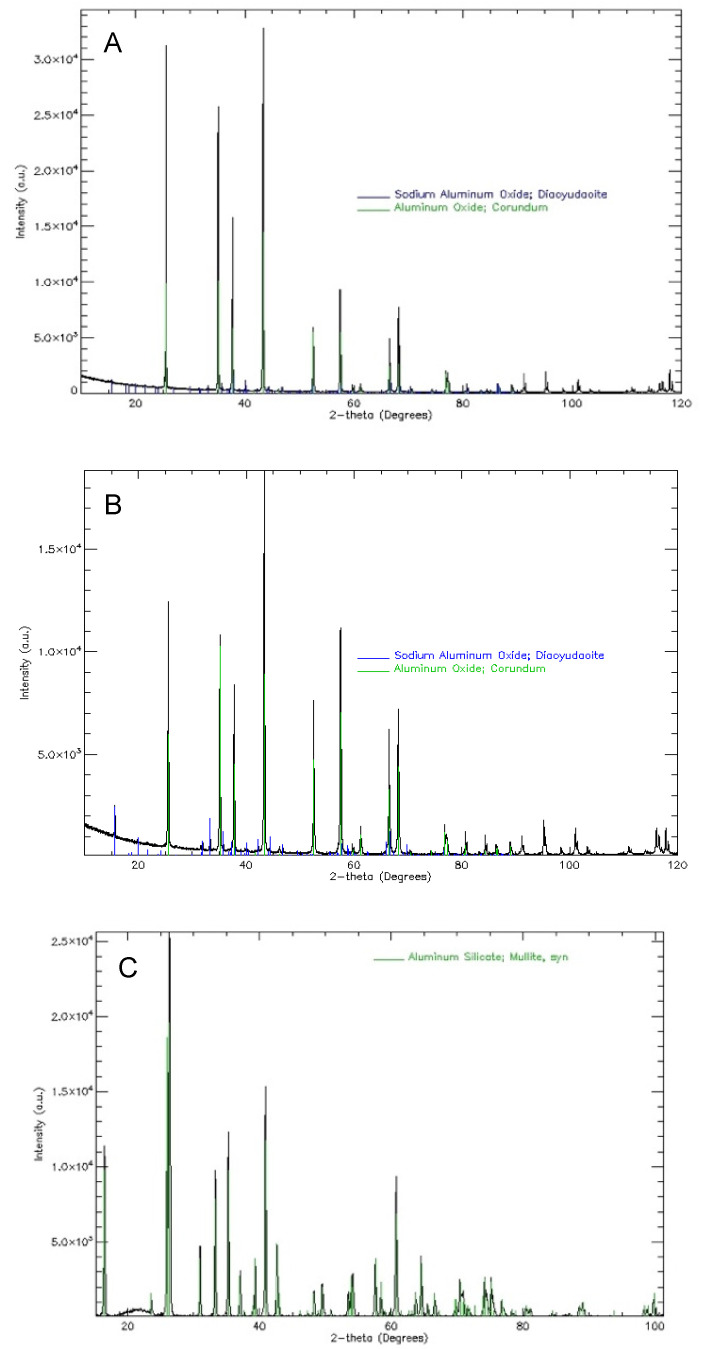
XRD diffractograms: (**A**) Al_2_O_3_ 200#; (**B**) Al_2_O_3_ 325#; and (**C**) Molochite 120.

The results of phase composition tests are presented in Figure 4 in the form of diffractograms. It should be emphasised that the tested Al_2_O_3_ 200# (Figure 4A) and 325 # powders (Figure 4B) powders are primarily characterised by a high content of α oxides. Analysis of the phase composition of the tested powders (Figure 4A–C) showed that they are single-phase materials. Analysis of the phase composition of the Molochite 120 powder (Figure 4C) showed that it is a single-phase material, composed only of Al_6_Si_2_O_13_ aluminosilicate-mullite. Furthermore, the diffractogram of Molochite 120 powder (Imerys, Cornwall, UK) shows an effect caused by the presence of the amorphous phase.

The selected powders are consistent in terms of chemical composition and particle size with the technological regime of industrial precision foundries.

### 3.2. Moulding Binders

Research was carried out on the selection of the optimal binder for the production of a ceramic mould for four types of binders used to produce sets of ceramic moulds. The first set, currently used, consists of a binder for the first layers, Ludox SK (contains colloidal silica), and a binder for the construction layers, hydrolysed ethyl silicate (HES). The set developed for new water-soluble binders, eliminating the HES binder from mass production, consists of a binder for the first layer, Keysol from Ransom and Randolph (Maumee, OH, USA), and a binder for construction layers, Matrixsol from Ransom and Randolph (Maumee, OH, USA).

Keysol is a two-component water-soluble binder, a concentrate based on an acrylic-vinyl copolymer with a weight content of 20–40% and the main aqueous solution containing SiO_2_ nanoparticles. Matrixsol is a two-component water-soluble binder, a concentrate of 2,2′,2″-(hexahydro-1,3,5-triazin-1,3,5-triyl)triethanol with a content of 70–100% by weight and a main aqueous solution containing SiO_2_ nanoparticles.

The research was based on the analysis of the properties of these binders, analysis of the impact on the environment (two water-based binders (K + M) including Keysol for the primary layer and Matrixsol for the backup layers), and determining whether they can replace existing standard binders based on ethyl alcohol. The main criterion for selecting these types of binders was to investigate which of these binders would be able to replace HES. The use of water-based binders is an interesting issue because they are more ecological materials and definitely more healthy for people working in the foundry. The use of binders and alcohol systems is harmful to the human body, causing dryness of the skin and conjunctiva. Replacement of HES with water-based binders will improve work safety, be more environmentally beneficial, and also create an opportunity to obtain moulding systems with better physical and mechanical properties compared to alcohol-based systems.

**Figure 5:**
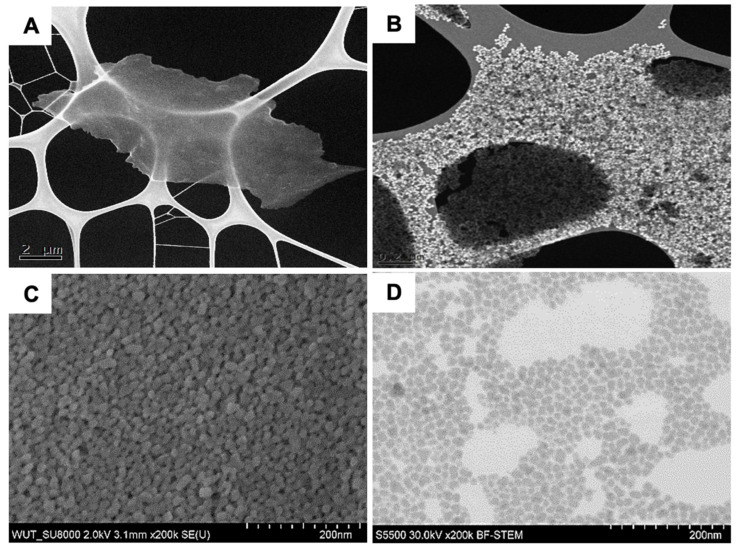
SEM images of binder particles: (**A**) HES; (**B**) Ludox SK; (**C**) Matrixsol; and (**D**) Keysol.

To characterise the nanoparticles present in the tested binders, SEM tests were performed, the results of which are presented in Figure 5. No nanoparticles were observed in the HES binder (Figure 5A). Analysis of the remaining binders showed the presence of SiO_2_ oxide particles in the dried samples. They were characterised by a shape close to spherical (Figure 5B–D). This is an important property of colloidal silica-based binders. When the ceramic moulds are dried, the binder undergoes the sol → gel transformation by the evaporation of water. As a result of the reaction between hydroxyl groups on the silica surface, SiO_2_ oxide combines into three-dimensional structures (agglomerates) through –Si–O–Si– bonds. The reaction that takes place is irreversible. Thanks to this process, the ceramic mould has good strength properties.

**Figure 6:**
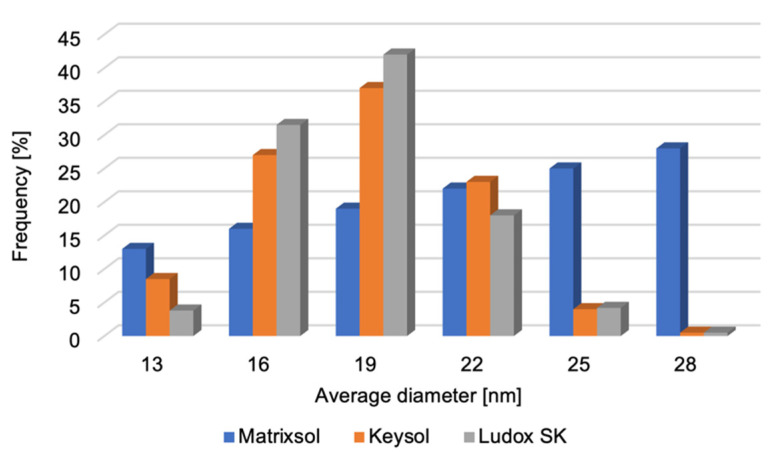
Distribution of the average particle diameter of the tested binders: Matrixsol, Keysol, and Ludox SK.

Measurements of the average particle diameter of the tested moulding binders were performed (Figure 6). Because of the inability to determine the presence of nanoparticles in the HES binder, no measurement was performed for it. It was found that the nanopowders are characterised by a unimodal distribution of average diameter. The small difference between the minimum and maximum diameters indicates the high uniformity of the SiO_2_ nanoparticles. Based on the analysis of microscopic examinations, it was shown that the SiO_2_ nanopowder particles included in polymer binders have a spherical shape. The average diameter of all nano-SiO_2_ particles does not exceed 18 nm. It should be noted that the study was conducted in an aqueous environment in which the agglomerates were dissolved. This makes it possible to determine the average diameter of the nanoparticles.

**Figure 7:**
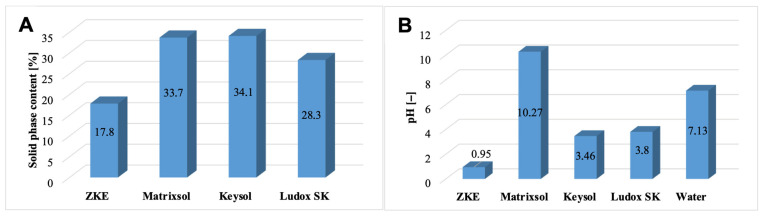
(**A**) Solid-phase content; (**B**) pH of the tested binders.

The solid-phase content in the tested moulding binders was also determined (Figure 7A). The HES binder has the lowest solid-phase content. Water-soluble binders are characterised by a high solid-phase content in the form of colloidal silica >28% weight. It is almost twice as large compared to HES. However, Keysol binders have the highest nano-SiO_2_ content, approximately 34.1% of weight, and Matrixsol, 33.7% of weight.

**Figure 8:**
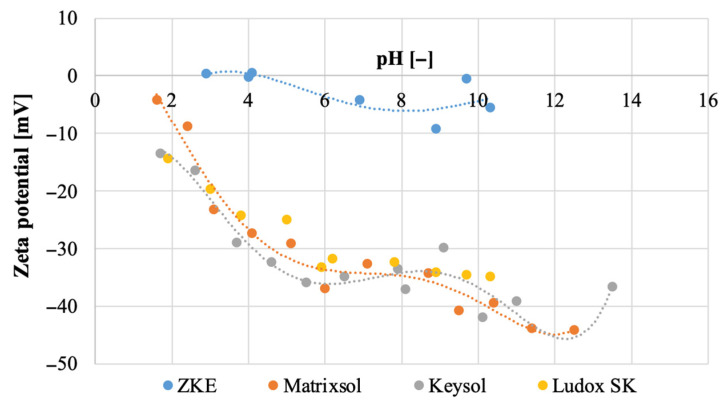
Zeta potential of the tested binders: Matrixsol, Keysol, and Ludox SK.

It was also established that the Matrixsol, Keysol, and Ludox SK binders differ significantly in terms of pH (Figure 7B). The reference HES binder is characterised by a strong acidic reaction, pH = 0.95. The keysol binder (pH = 3.46) and the Ludox SK (pH = 3.80) are less acidic. However, the Matrixsol binder has an alkaline reaction with a pH = 10.27. Analysis of the potential values of the tested binder species showed that the reference HES binder has an isoelectric point at pH = 4.4 (Figure 8). For pH > 4.4, the potential is negative and has a minimum value of −9.2 mV. Keysol, Matrixsol, and Ludox SK binders have a negative potential in the entire pH range ranging from −13.5 mV for pH = 1.7 to −41.9 mV for pH = 10.1; −4.25 mV for pH = 1.6 to 44.2 mV for pH = 12.5; and −14.3 mV for pH = 1.9 to −34.9 mV for pH = 10.3. These binders for pH > 4 show a high electrokinetic stability of the dispersion. Binder particles have a high force that causes them to repel each other; there is no tendency to form agglomerates [18].

**Figure 9:**
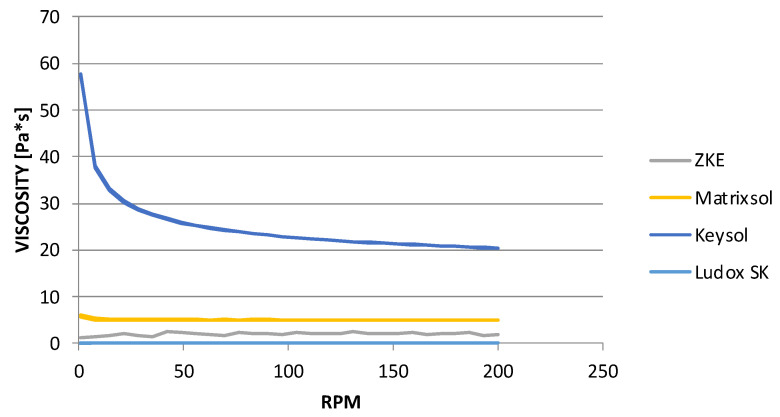
Dynamic viscosity of the tested binders: Matrixsol, Keysol, and Ludox SK.

At the same time, the results of dynamic viscosity tests (Figure 9) showed that the binders, Matrixsol and Ludox SK, belong to the rheologically unstable group. They are characterised by a low viscosity, and this is one of the reasons for their irregular characteristics. The second may be the inhomogeneity of the tested samples. The suspension could have sedimented during the preparation of the test. It can be stated that Matrixsol and Ludox SK binders belong to Newtonian fluids; their viscosity does not depend on shear. The highest viscosity is observed for the Keysol binder, which belongs to the group of pseudoplastic liquids; it thins out as the shear rate increases. It was also determined that increasing the spindle speed leads to a reduction in the viscosity of the Keysol binder from 57 to 20 mPas, that is, almost three times.

**Figure 10:**
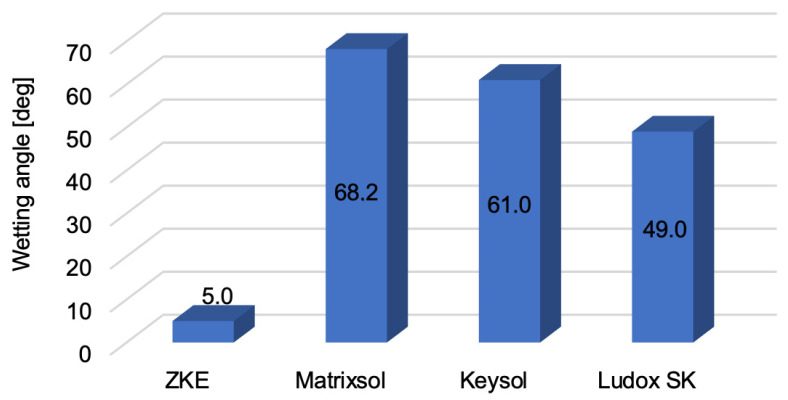
Wetting angle of the tested binders: HES, Matrixsol, Keysol, and Ludox SK.

Analysis of the results of the wetting angle test (Figure 10) showed that the binders have different wetting angle values. For HES, the wetting angle is 5 degrees. It can be assumed that this binder is a liquid that wets the surface well. Matrixsol, Keysol, and Ludox SK binders are characterised by hydrophobic properties.

**Figure 11:**
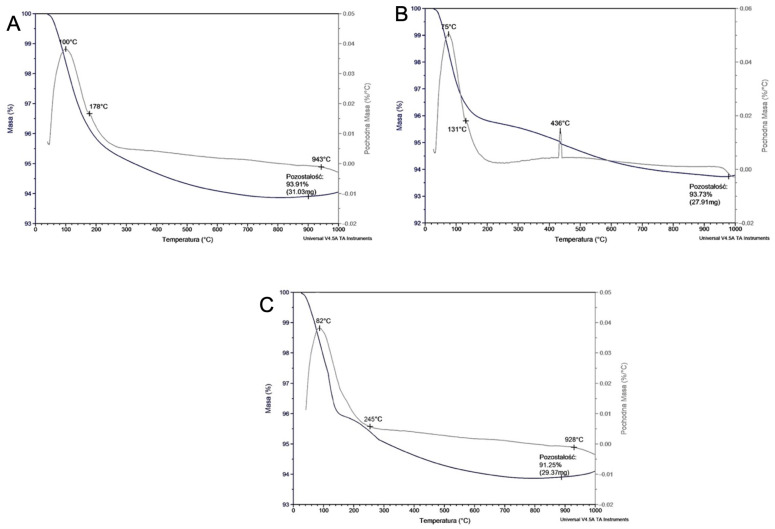
Thermogravimetric curves of the binder: (**A**) Matrixsol, (**B**) Keysol, and (**C**) Ludox SK.

Thermogravimetric analyses of the Matrixsol, Keysol, and Ludox SK (Figure 11A–C) binders were performed. TGA curves show a rapid mass loss for all binder grades up to a temperature of approximately 190 °C. This causes the water to evaporate quickly. Subsequent peaks are characteristic of the decomposition of polymer compounds, components of the tested binders. According to the literature [11,12,13], these values correspond to these substances with a slight change in temperature, probably caused by the presence of impurities or additives that help increase the stability of the binders.

### 3.3. Moulding Sands

**Figure 12:**
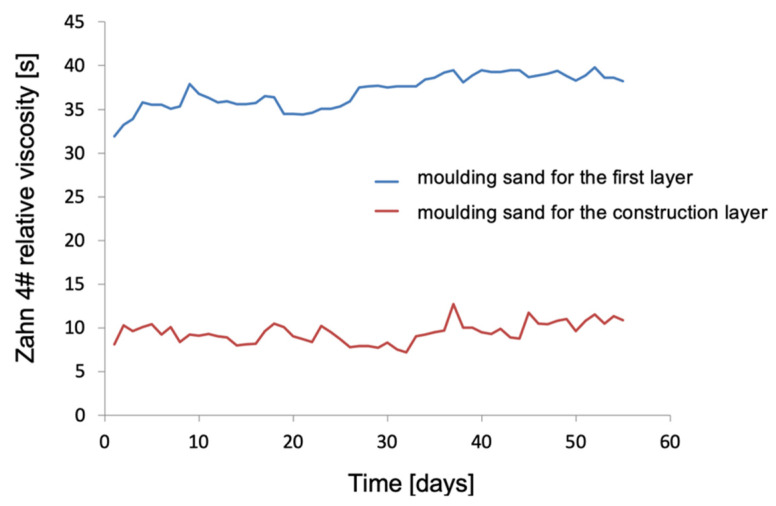
Zahn 4# relative viscosity of the moulding sand for the first layer and the construction layers as a function of time.

The moulding sands were obtained after mixing their components in appropriate proportions: binder, matrix powders, and wetting and antifoam additives. The prepared mass was mixed using a mechanical mixer under strictly defined conditions of temperature, humidity, and air circulation in the room. They have a specific content of powders, binders, and antifoam and wetting additives. The first moulding sand was prepared from Al_2_O_3_ powder with two gradations 200# and 325# in the 50–50% ratio. Keysol was the main binder. Analysis of the results of the moulding sand test for the structural layers (Figure 12) confirms its high stability during 8 weeks of testing.

It should be noted that the relative viscosity of the ceramic mass for the model layer is clearly higher than that of the moulding mixture intended for the structural layers. However, these ranges correspond to the actual times that the fluid flows from the draw-off cup. It is assumed that for pourable masses intended for the first layer, the flow time should be 10–20 s, while for mixtures for construction layers it should be 25–40 s. The results obtained show that both moulding sands meet the assumptions. For this reason, both moulding sands were used to obtain mould samples.

### 3.4. Ceramic Moulds

The lack of sufficient data in the literature regarding the forming method, as well as the properties that the ceramic mould should have, and therefore the finished casting, was the basis for a comprehensive analysis of the process of producing ceramic moulds using hydrolysed ethyl silicate. The test results obtained make it possible to determine the predicted properties of the mould produced in the process using new water-soluble binders.

Multilayer ceramic moulds were made by applying successive layers to a wax model. The model was immersed in a ceramic mass and sprinkled with coarse ceramic powder. The prepared layer was left to dry under established conditions of temperature, humidity, and time. These activities were repeated until seven layers were created. The first two layers were made of a higher-viscosity moulding sand for better coverage and reproduction of the surface of the wax model. However, the structural layers were made of a lower-viscosity mass. The ceramic mould was then heat treated to remove wax and increase its mechanical strength. This process consisted of three stages:

- melting wax—in an autoclave, in an atmosphere of superheated steam at a temperature of 170 °C and a pressure of 0.86 MPa;
- wax firing—in an electric furnace, at a temperature of 760 °C, for 1 h, cooling with the furnace;
- annealing the mould—in an electric chamber furnace, at a temperature of 1200 °C, for 2 h, cooling with the furnace.

Ceramic moulds (marked K + M) were made of Al_2_O_3_ + Keysol moulding sand for the first layer and Molochite 120 + Matrixsol for the construction layers, with controlled air circulation, humidity, and temperature conditions. The conditions used made it possible to reproduce the process of producing ceramic moulds using the previously used HES binder. The inability to dry experimental forms in an industrial dryer led to the development of other evaluation methods. The test results obtained for both the HES form and the newly developed form were the basis for a comparative analysis.

**Figure 13:**
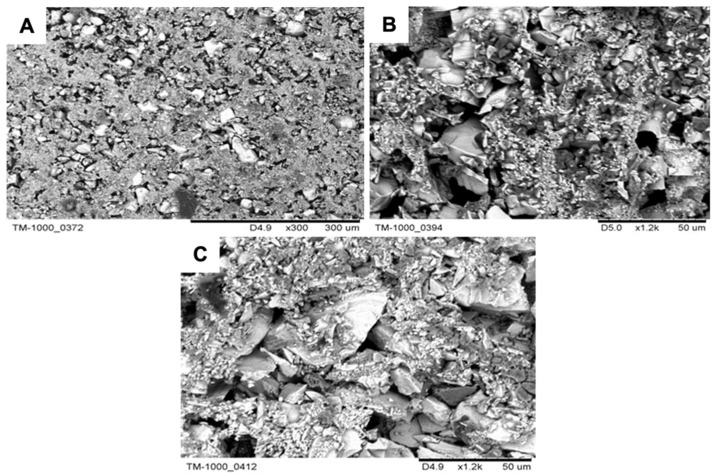
Images of the surface of the first layer of the HES mould: (**A**) raw state; (**B**) after firing the wax 760 °C/1 h; and (**C**) after annealing at 1200 °C/1 h.

**Figure 14:**
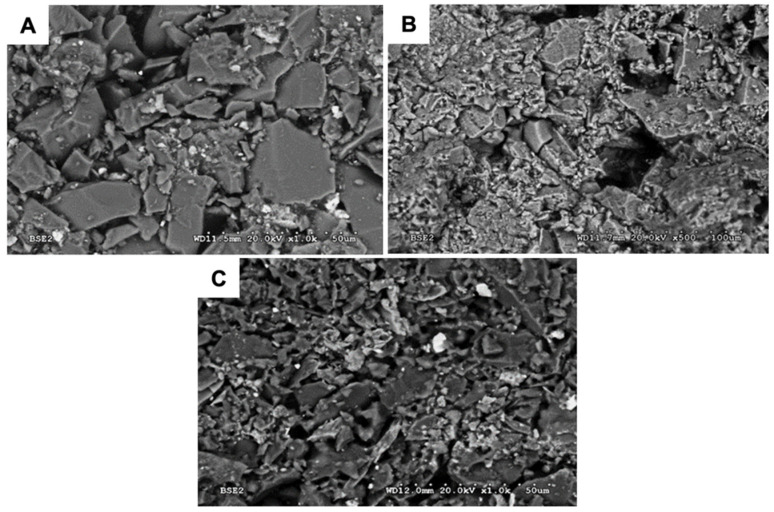
Images of the surface of the first layer of the K + M mould: (**A**) raw state; (**B**) after firing the wax at 760 °C/1 h; and (**C**) after annealing at 1200 °C/1 h.

Microscopic examinations allowed the characterisation of the geometric structure of the HES (Figure 13) and K + M (Figure 14) ceramic moulds in three states: raw, after wax firing at 760 °C/1 h, and after annealing at 1200 °C/1 h. It was found that the surface of the tested forms was non-uniform and porous. Particles of moulding powders with sharp edges used as a matrix were observed. The fractures obtained were brittle. Increasing the temperature of heat treatment resulted in an increase in the number of pores.

Analysis of the phase composition of the mould material after the process of melting and firing the wax mould model showed the presence of phase components:

- α Al_2_O_3_ oxide—matrix and topping of the first and second layers;
- Al_2_O_3_·SiO_2_ mullite—matrix of the structural layers of the mould;
- sillimanite.

Additionally, a low content of α cristobalite was found in the annealed forms. In the process of annealing the mould material, a phase transformation of amorphous SiO_2_ oxide occurs, which is a component of the binder and Molochite 120 powder. It should be noted that the relative volume of this phase component is small. Its presence is not important for technological reasons. However, crystalline SiO_2_ oxide, especially cristobalite, is dangerous to human health. Therefore, it is recommended to be particularly careful when creating the moulds. The tests also confirm the good thermal stability of the main materials used in the moulding of sands, corundum, zirconium silicate, cobalt aluminate, and mullite. This applies to all stages of the heat treatment of ceramic moulds.

**Figure 15:**
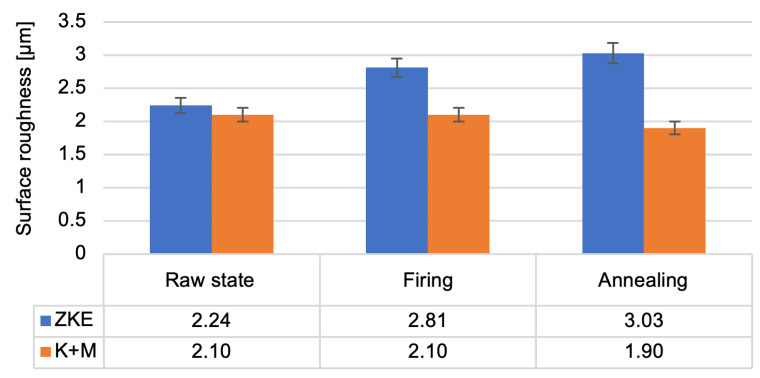
Surface roughness of the HES and K + M moulds in the raw state, after firing at 760 °C/1 h, and after annealing at 1200 °C/1 h.

Figure 15 shows the average surface roughness of the HES and K + M moulds. Moulds that use the HES binder are characterised by an increase in the Ra parameter as a result of heat treatment. However, the moulds developed and manufactured using water-soluble binders show similar values of average surface roughness for samples in all states, i.e., in the raw state, after firing, and after annealing. The low Ra values are influenced by silica nanoparticles that fill the pores open at the surface. For aviation applications, Ra should not exceed 5 µm. As can be seen from the results obtained, the developed moulding systems meet the requirements. The silica nanoparticles (SiO_2_) present in water-soluble binders such as Keysol and Matrixsol act as fillers by entering microvoids and surface irregularities during the drying and sintering stages. Upon gelation and subsequent thermal treatment, they form a continuous network that seals surface pores and reduces roughness. This results in a smoother surface finish, which is particularly advantageous for aerospace casting applications.

**Figure 16:**
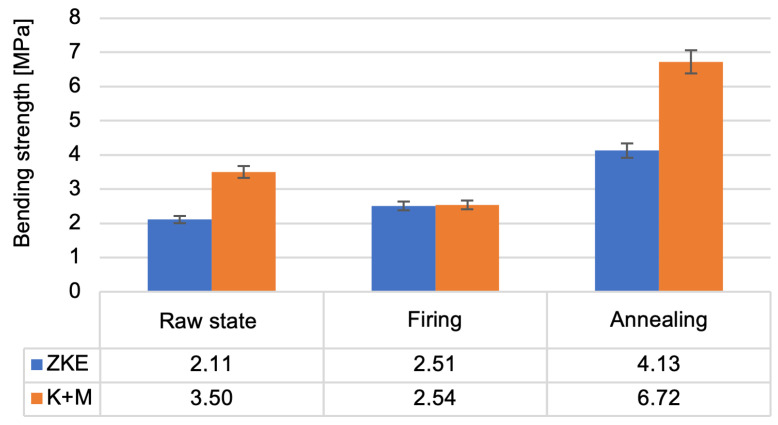
The bending strength of the K + M and HES mould material in the raw state, after wax firing at 760 °C/1 h, and after annealing at 1200 °C/1 h.

**Figure 17:**
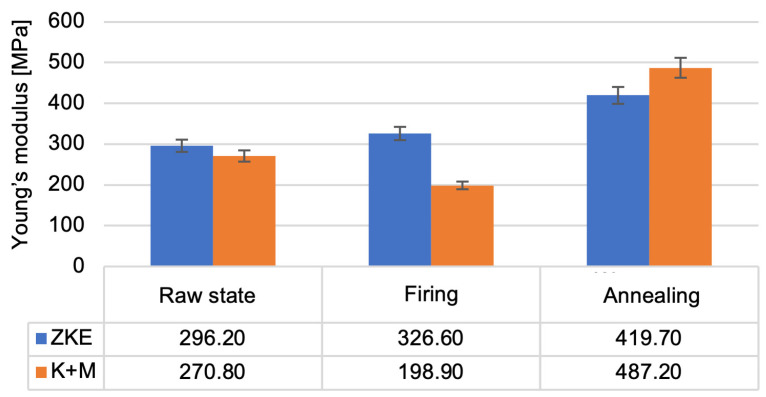
Young’s modulus of the K + M and HES mould material in the raw state, after wax firing at 760 °C/1 h, and after annealing at 1200 °C/1 h.

Analysis of the results of the testing of the mechanical properties of the created ceramic moulds showed that the highest bending strength (Figure 16) and the Young modulus value (Figure 17) were obtained after annealing. For bending strength, measurements were performed on ten specimens per group (HES and K + M) in three material states: raw, after wax firing (760 °C/1 h), and after final annealing (1200 °C/2 h). In the annealed state, the average bending strength for HES-based moulds was 11.3 MPa (±0.9 MPa), whereas the K + M moulds achieved 12.4 MPa (±0.7 MPa). A two-tailed Student’s *t*-test showed that this difference was statistically significant (*p* = 0.0037), indicating superior post-sintering mechanical integrity in the water-based system [19].

**Figure 18:**
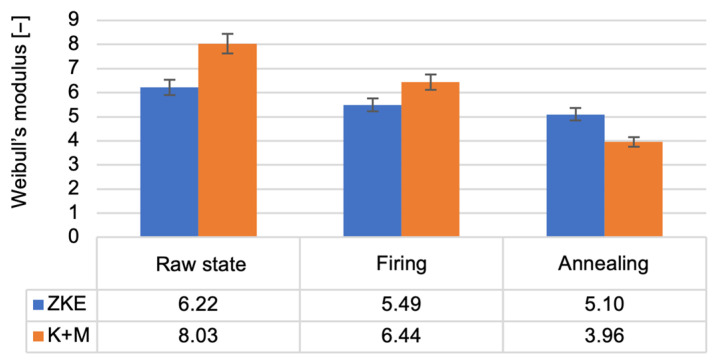
Weibull modulus of the K + M and HES mould material in the raw state, after wax firing at 760 °C/1 h, and after annealing at 1200 °C/1 h.

In the raw state, HES moulds exhibited slightly higher bending strength (8.7 MPa vs. 8.2 MPa), which is attributed to the strong initial cohesion provided by the alcohol-based binder [21]. However, this difference was not statistically significant (*p* = 0.087), and the values converged after heat treatment. Notably, K + M moulds demonstrated better thermal resilience, retaining higher mechanical performance after each processing stage, consistent with findings in recent work on water-based ceramic slurries [16,24]. The reduction in bending strength after wax firing is caused by the decomposition of the polymer contained in the binder; it provides greater bending strength in the raw state. The increase in bending strength after annealing is related to the sintering process of the ceramic matrix and colloidal silica. The value of the Weibull modulus (Figure 18) decreases as the annealing temperature of the ceramic moulds increases. This is due to the high porosity of the samples, which resulted in low Weibull modulus values for all samples. The m modulus for the K + M samples is smaller than that for the HES samples, which could be due to the lower value of the wetting angle of the hydrolysed ethyl silicate, which resulted in good mixing of the composition and a smaller number of surface defects.

In terms of porosity, mercury intrusion porosimetry revealed that the average open porosity in annealed HES moulds was 26.8%, while in K + M moulds it was slightly higher at 29.1%. Although this increase may appear unfavorable at first glance, further analysis showed that the gas permeability of K + M moulds was significantly enhanced—5.6 × 10^−9^ cm^2^ at 1100 °C versus 4.1 × 10^−9^ cm^2^ for HES moulds—leading to improved venting during casting [13,25]. This effect is aligned with the beneficial role of porosity in degassing described in advanced investment casting systems [17,20]. The increased porosity in the K + M system also had no measurable negative impact on casting surface quality, as evidenced by the Ra values (<5 µm) and the absence of gas-related defects in pilot trials.

For surface roughness, the average Ra of K + M moulds after annealing was 4.2 µm, compared to 5.1 µm in the HES group. This difference was statistically significant (*p* = 0.012), confirming that silica nanoparticle infiltration in the K + M binder contributed to improved surface quality by partially sealing surface pores during drying and sintering [12,14].

The improved mechanical, thermal, and surface characteristics of the K + M system strongly support its practical replacement of the traditional HES-based solution, in line with sustainable material trends described in the recent literature [16,26,27].

**Figure 19:**
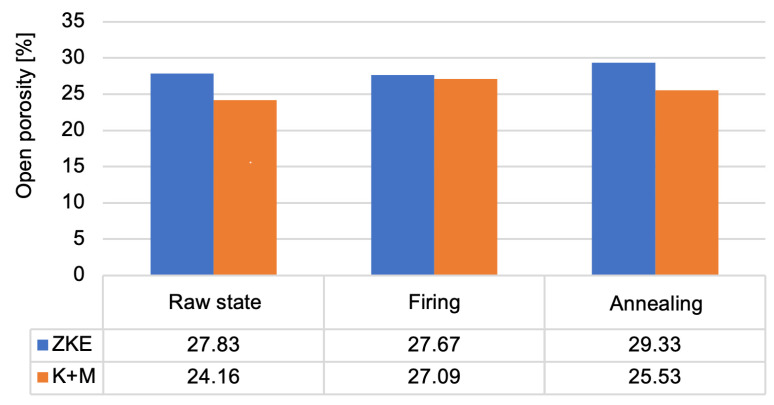
Open porosity of the K + M and HES mould material in the raw state, after wax firing at 760 °C/1 h, and after annealing at 1200 °C/1 h.

**Figure 20:**
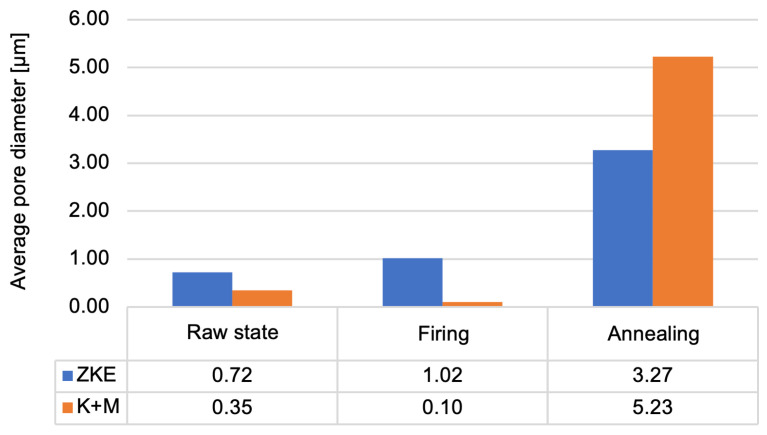
Average pore diameter of the K + M and HES moulds in the raw state, after wax firing at 760 °C/1 h, and after annealing at 1200 °C/1 h.

Analysis of the results obtained from the porosity test (Figure 19) allows us to conclude that the porosity of the moulds ranges from 24% to 29%. Annealed forms are characterised by the highest porosity. This is caused by the sintering process. Up to a temperature of 760 °C, the entire binder does not undergo thermal decomposition. More is decomposed at 1000 °C, which also results in greater porosity. This parameter is also influenced by the rapid sintering process of 10 °C/min, which causes faster gas escape, the formation of voids, and the inability to close pores. Wax firing and annealing cause the evaporation of water and the degradation of organic compounds. After annealing, the average pore diameter increases significantly (Figure 20) in the tested ceramic moulds. This proves the coalescence of pores and the sintering of the fine-grained fraction of the matrix ceramic powders. This is also evidenced by the distribution of the average pore diameter. The annealing process eliminates the occurrence of small pores. The high porosity of the mould in the annealed condition and the large average pore diameter are advantageous. Porous ceramic moulds are characterised by good gas permeability when poured with liquid metal and are also brittle when finishing castings. Figure 20 shows that, in the case of the K + M system, the fired form has the highest porosity. For the K + M moulds, firing at 760 °C does not fully decompose organic additives, and partial sintering begins only after annealing at 1200 °C. Thus, larger pores are formed during annealing due to extensive sintering and agglomeration of ceramic particles.

The increased porosity observed in the K + M moulds, particularly after annealing (up to ~29.1%), indeed plays a dual role in the performance of the ceramic-casting system. On one hand, higher open porosity enhances gas permeability, which is a critical factor in avoiding casting defects such as gas porosity, blowholes, and oxide inclusions during metal pouring [13]. This is especially beneficial in the casting of nickel superalloy components, where complete and rapid evacuation of air and residual volatiles through the mould wall is essential to ensure high surface quality and structural integrity of thin-walled blades [4,16,24]. The gas permeability of K + M moulds increased with temperature, reaching a peak value of 5.6 × 10^−9^ cm^2^ at 1100 °C, compared to 4.1 × 10^−9^ cm^2^ for the HES system, which demonstrates superior outgassing capability [13]. This enhanced permeability, in combination with good wettability (moderate wetting angles) and phase stability, contributed to the absence of internal defects or misruns in pilot castings, confirming the functional viability of the developed solution. On the other hand, excessive or uncontrolled porosity may compromise surface finish or reduce local mechanical strength of the mould, particularly in areas of high thermal gradient or metallostatic pressure [20,27]. Surface roughness may increase due to micro-cavities formed during binder burnout and sintering, especially if the pore structure is irregular or contains interconnected macrovoids. However, in our tests, surface roughness (Ra) remained below the critical threshold of 5 µm, and no crack propagation or surface degradation was observed after annealing or during pilot casting trials.

To ensure control over porosity and mitigate potential negative effects, the following strategies should be applied:

1. Optimization of binder-to-powder ratio to reduce excess binder phase and control pore evolution during drying and sintering;
2. Use of fine silica nanoparticle-rich sealant coatings for the final layer (without dusting), which partially closes surface pores and minimizes Ra increase during firing [12,14];
3. Tuning of the sintering schedule, e.g., by incorporating a pre-sintering step at 900–1000 °C to stabilize the pore structure before final densification at 1200 °C;
4. Inclusion of pore modifiers or sacrificial pore-forming agents (e.g., fugitive organics or microballoons) that decompose at lower temperatures, leading to more uniform porosity distribution [17,25];
5. Post-casting leaching or finishing processes, such as water jet or mechanical polishing, to eliminate residual ceramic that may have penetrated into micro-defects.

Overall, while increased porosity is an inherent consequence of using water-based systems with high SiO_2_ content, it has been managed effectively through material and process design. The resulting moulds maintained both functional integrity and surface quality comparable or superior to conventional HES-based systems, which supports their practical implementation in aerospace casting technologies [27].

**Figure 21:**
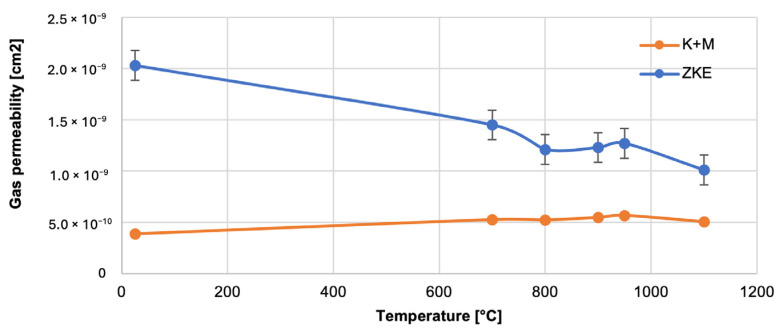
Gas permeability of the K + M and HES mould material in the temperature range from 22 °C to 1100 °C.

The results of the gas permeability tests of the tested ceramic moulds are shown in Figure 21. An increase in gas permeability with increasing temperature was found for the ceramic moulds using a water-soluble binder. However, for forms that used HES, the opposite phenomenon was observed. This is caused by the difference in the linear expansion coefficient of the mould ceramic material. The reduction in gas permeability at temperatures > 1100 °C is caused by the effect of sintering and shrinkage of the ceramic metal.

**Figure 22:**
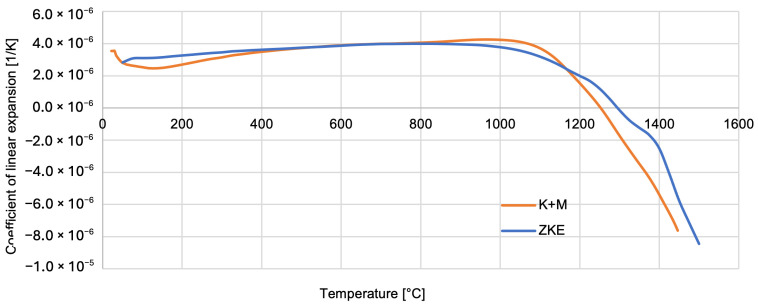
Coefficient of linear expansion of the K + M and HES mould material in the temperature range from 22 °C to 1500 °C.

**Figure 23:**
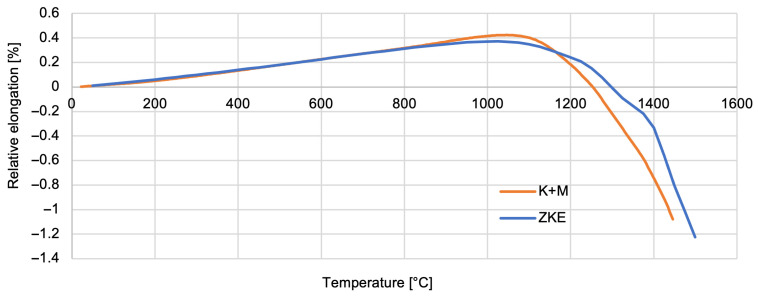
Relative elongation of the K + M and HES mould material in the temperature range from 22 °C to 1500 °C.

Thermal properties of the reference mould material were tested using HES, and that of the newly developed one was tested using K + M. The analysis of the results obtained indicates that the relative elongation increases slightly and reaches a maximum at a temperature of approximately 1100 °C—0.5% (Figure 22). Above the temperature of 1100 °C, the mould material shrinks to a maximum value of 1.5%. It is caused by the ceramic sintering process. Similar dependencies were found for the linear expansion factor of the material of the tested forms (Figure 23).

**Figure 24:**
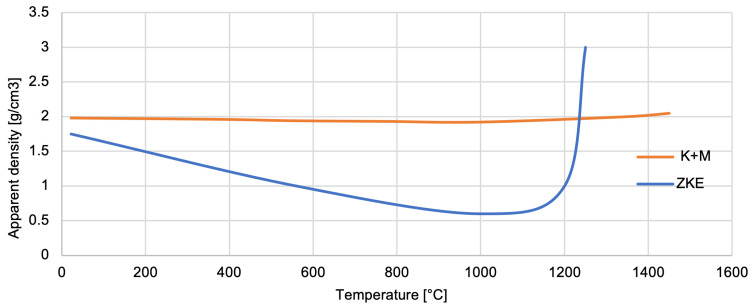
Apparent density of the K + M and HES mould material in the temperature range from 22 °C to 1500 °C.

Analysis of the measurement of the apparent density of the mould material (Figure 24) shows that it decreases as the temperature increases to its critical value—approximately 1100 °C. The formation of new phases then causes the material to thicken and increase its density [17]. The density increase after phase transformation and sintering improves mould mechanical strength and thermal shock resistance. A denser mould reduces gas entrapment and enhances casting accuracy. However, excessive densification could limit gas permeability. In the K + M system, we observed an optimal balance, leading to improved mould integrity without sacrificing permeability.

**Figure 25:**
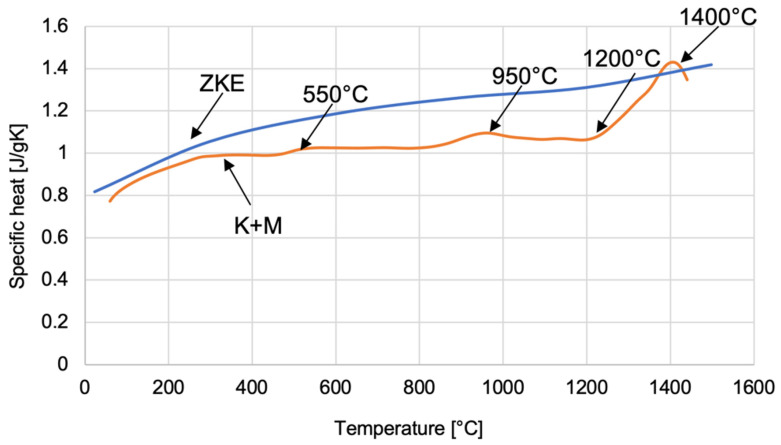
Specific heat of the K + M and HES mould material in the temperature range from 22 °C to 1500 °C.

The dependence of the specific heat on temperature was determined for ceramic mould materials using HES and K + M binders (Figure 25). The curve obtained allows one to determine four characteristic temperature values related to the polymorphic transformation of SiO_2_ oxide. At a temperature of approximately 550 °C, α-quartz transforms into the β variety. Then, at a temperature of approximately 960 °C, the transformation of β-quartz → β-tridymite takes place. The β-quartz variety is transformed into β-cristobalite at a temperature of 1200 °C. However, at a temperature of approximately 1400 °C, β-tridymite → β-cristobalite. The specified polymorphic transformation temperatures differ slightly from the theoretically determined polymorphic transformation temperatures of SiO_2_ oxide. This is caused by the presence of various chemical additives in SiO_2_, which shift the temperatures of the polymorphic transformation.

**Figure 26:**
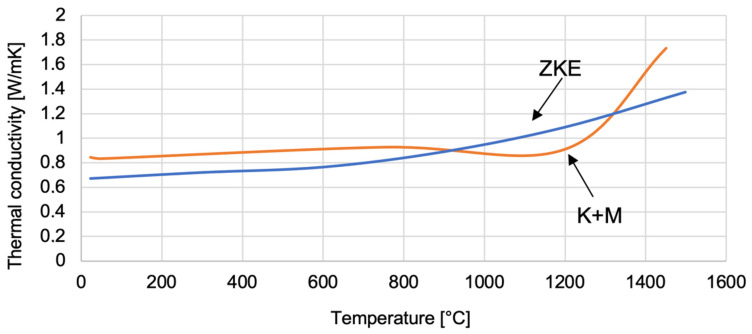
Thermal conductivity of the K + M and HES mould material in the temperature range from 22 °C to 1500 °C.

**Figure 27:**
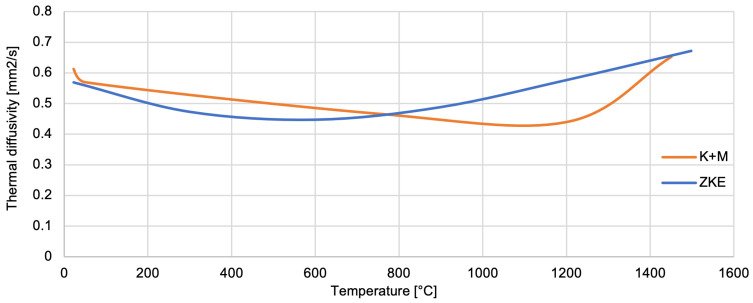
Thermal diffusivity of the K + M and HES mould material in the temperature range from 22 °C to 1500 °C.

The thermal conductivity of SiO_2_ oxide was determined (Figure 26). The thermal conductivity coefficient ranges from 0.3–1 W/mK up to a temperature of approximately 1100 °C. The thermal diffusivity of SiO_2_ oxide also decreases from 0.62 mm^2^/s to 0.43 mm^2^/s, also up to a temperature of 1100 °C (Figure 27). SiO_2_ oxide is characterised by low conductivity and thermal diffusivity due to high porosity. A particularly important factor is increasing the temperature value to 1100 °C. It causes the sintering effect of ceramics, shrinkage, and thickening of the mould material, hence the increase in thermal conductivity to 2 W/mK and thermal diffusivity to 0.67 mm_2_/s at a temperature of 1400 °C. The analysis of the results of the thermal properties test indicates significant changes that are caused by the polymorphic transformation characteristic of the SiO_2_ oxide. A particularly large difference in values was found at a temperature of approximately 1100 °C, which is the beginning of the ceramic sintering process. In addition, at this temperature, large changes in relative elongation, increased density, and thermal conductivity of the mould material were demonstrated. This indicates both shrinkage and compaction of the mould material. Additionally, characteristic values of the temperature of the polymorphic transformation of SiO_2_ oxide can be distinguished in the curves of specific heat versus temperature.

In terms of binder systems, our findings support and extend previous work by Ismael et al. [17], who investigated colloidal silica as a nanostructured binder for refractory castables. While they confirmed the potential of colloidal systems for improved thermal stability, our study goes further by integrating a dual-component water-soluble binder system (Keysol + Matrixsol) and demonstrating its full industrial viability for multilayer ceramic moulds in aerospace applications. Similarly, Wang et al. [24] studied ceramic shell–metal interactions and gas permeability in yttria-based moulds; our work complements theirs by focusing on silica-alumina-based moulds but introduces a comparable porosity–permeability balance critical for thin-walled casting. Regarding mechanical properties, our annealed K + M moulds achieved bending strengths exceeding 12 MPa, which are higher than the values reported in earlier work by Maity and Maity [14], where ceramic moulds reinforced with zircon/silica layers reached strengths of only 8–10 MPa under comparable heat treatment conditions. Furthermore, our observed Young’s modulus and Weibull modulus trends are consistent with the mechanical behavior described by Donachie et al. [18] in superalloy-compatible mould systems, yet we extend this by correlating these values with binder microstructure (e.g., nanoparticle dispersion and sol-gel kinetics). In terms of thermal behavior, our thermal conductivity results (rising from 0.3 to 2.0 W/mK with temperature) align with those obtained by Szeliga et al. [13], who measured similar trends in investment casting shell moulds. However, unlike their work, we incorporate a detailed thermal diffusivity analysis and correlate it with porosity and phase transformations (e.g., quartz → tridymite, cristobalite transitions) observed via DSC, thus offering a more complete thermophysical characterisation. Concerning surface quality, our surface roughness (Ra < 5 µm) after sintering matches the performance benchmark established by Chen et al. [27] for alumina-based, laser-fused ceramic shells, despite the fundamentally different forming method. Our study thus demonstrates that water-based slurry systems can match or outperform more technologically intensive fabrication methods when appropriately optimized. From an environmental perspective, Cunha et al. [27] demonstrated the feasibility of using waste ceramic moulds and paraffin wax in mortar composites, highlighting the lifecycle aspect of foundry materials. Our work contributes to this direction by proving that alcohol-free binder systems not only reduce VOC emissions by over 85% but also generate safer solid waste for downstream recycling, in line with circular economy goals. Finally, the present work is, to the best of our knowledge, the first comprehensive experimental validation of the Keysol/Matrixsol system for precision aerospace moulds that includes mechanical, thermal, microstructural, and gas permeability performance comparisons against conventional HES systems—thereby closing an existing gap in the literature on water-based binder substitution under production-simulating conditions [27].

### 3.5. Environmental and Economic Benefits

The environmental impact of HES-based binder systems is primarily associated with the evaporation of alcohol solvents (e.g., ethanol and isopropanol), which release large quantities of volatile organic compounds (VOCs) during application and drying. According to industry data and environmental audits, the use of HES binders typically results in the emission of 450–600 g VOC per kilogram of binder used. For a standard seven-layer ceramic shell system with a total binder requirement of approximately 2.5 kg per mould, this translates to 1.1–1.5 kg of VOCs emitted per unit. In contrast, the Keysol and Matrixsol systems are water-based and contain no organic solvents, effectively reducing VOC emissions by over 90% [28]. Evaporation of water during drying is slower, but environmentally neutral, and does not require additional air treatment or filtration systems. As a result, foundries adopting the K + M system can eliminate the need for VOC capture and incineration systems, reducing energy consumption and lowering their total carbon footprint. In terms of greenhouse gas (GHG) emissions, a lifecycle comparison based on preliminary process modeling (including transportation, energy for drying, and waste treatment) indicates that switching from HES to K + M binders can reduce the total CO_2_ equivalent emissions per mould by approximately 18–25%, depending on the region’s energy mix and drying strategy [29,30]. This is particularly relevant for companies certified under the EU Emissions Trading Scheme, where measurable reductions in Scope 1 and Scope 2 emissions are critical. Another important factor is workplace safety and exposure. HES systems are classified as flammable and hazardous under REACH and GHS regulations. Ethyl silicate can irritate the respiratory tract and mucous membranes, especially in high-humidity environments, requiring the use of fume hoods, explosion-proof ventilation, and personal protective equipment (PPE). In contrast, Keysol and Matrixsol have no flash point, produce no hazardous fumes, and are classified as non-toxic under standard occupational exposure thresholds. This contributes to significantly improved health and safety conditions in the workplace, fewer incidents of exposure, and reduced risk of fire or explosion, especially during summer operation or in poorly ventilated areas. From an economic standpoint, the use of water-based binders may initially appear more expensive on a per-liter basis. However, a full cost-benefit analysis shows that the total operational cost per mould is in fact lower when using K + M, once indirect costs such as solvent handling, fire safety infrastructure, VOC permits, waste disposal, and equipment maintenance are included. In addition, the use of aqueous systems enables better automation of slurry application and cleaning, leading to improved process repeatability and shorter setup times. Several industrial partners reported a net reduction of approximately 10–15% in total moulding-related costs after converting to water-soluble binder systems, despite a slightly higher material unit price. Furthermore, the ecological nature of the K + M system offers additional strategic benefits. It improves the company’s compliance with international green production standards and can support environmental declarations such as Environmental Product Declarations (EPDs) or EcoVadis certifications. This is particularly advantageous in public tenders or international supply chains where sustainability criteria are increasingly weighted in supplier selection [31].

In summary, the replacement of HES with water-soluble binders such as Keysol and Matrixsol brings quantifiable environmental benefits, including a >90% reduction in VOC emissions, a 20–25% reduction in carbon footprint, elimination of flammable solvents, and enhanced occupational safety. Economically, it reduces long-term operational costs and aligns with both current and future legislative and market-driven sustainability requirements. These factors not only justify the adoption of water-based systems from a technical standpoint but also demonstrate their strategic advantage for modern foundries.

### 3.6. Pilot-Scale Industrial Testing

During the pilot tests, a series of ceramic moulds were produced using the full seven-layer K + M system. Each layer was applied under standard shop-floor conditions (ambient temperature ~22 °C and humidity ~50%), without the use of a high-temperature industrial drying oven, to simulate minimal infrastructure adaptation. The moulds were then subjected to the standard debinding and sintering processes—dewaxing in an autoclave at 170 °C, firing at 760 °C for 1 h, and final annealing at 1200 °C for 2 h—exactly replicating the conditions used for HES-based systems. The castings produced were hollow turbine blades from a IN713C nickel-based superalloy, which were chosen due to their complex geometry, thin-wall design, and high surface finish requirements. The K + M moulds demonstrated excellent dimensional stability and structural integrity during all stages of the process. No delamination, interlayer cracking, or deformation was observed, even under metallostatic pressures and high thermal gradients. The surface of the resulting castings exhibited a roughness (Ra) below 5 µm, within aerospace specification limits. Additionally, repeated casting cycles using the same slurry compositions and process parameters confirmed the process repeatability of the binder system. Over three production batches (each with 8–10 moulds), no batch-to-batch variation exceeding 5% was recorded in key quality metrics, including shell thickness, porosity, and casting surface fidelity. Importantly, workers involved in the pilot-scale trials reported improved handling characteristics and a reduction in unpleasant odours and skin irritation, which are commonly associated with alcohol-based binders such as HES. The use of water-based systems eliminated the need for explosion-proof equipment and reduced the frequency of ventilation system maintenance, further supporting the operational feasibility of the new solution. From a scalability standpoint, the slurry formulations and drying protocols used in the pilot tests were intentionally designed to be transferable to existing foundry infrastructure with minimal modification. The equipment used (slurry tanks, mixers, wax trees, and drying chambers) remained unchanged. This demonstrates that full-scale industrial adoption of the K + M system can be achieved without extensive investment in new hardware or process requalification.

In conclusion, the pilot-scale validation confirmed that the water-soluble binder system developed in this study is not only technically effective but also scalable, repeatable, and compatible with industrial production environments. The successful application in turbine blade casting—one of the most demanding precision casting tasks—provides strong evidence that the proposed system is ready for production implementation, offering environmental, safety, and economic advantages over traditional HES-based solutions.

## 4. Summary

The research and analysis performed were the basis for expanding our knowledge to the extent necessary to develop a new ecological technology of ceramic-casting moulds for the precise casting of aircraft engine turbine blades from nickel superalloys. The essence of the investigation was to eliminate a harmful factor (ethyl alcohol) from the process of preparing a ceramic mould. Fundamental differences in the technological properties of alcohol and water-based binders forced the development of new criteria for the selection of moulding sand components, including dustings, procedures for selecting moulding mixture components, production conditions, and control of the technological properties of moulding sands and forming and drying technologies, as well as the heat treatment of moulds and the process of pouring the liquid alloy itself. Analysis of the results of the comprehensive research and development work, both on a laboratory scale and on a production scale, showed that the stated goal was achieved.

The usefulness of water-soluble binders and a new type of water-based moulding system for obtaining ceramic-casting moulds in the aviation and foundry industries were found. It has been proven to be used in production. It has been proven that they can be used in production and, at the same time, excluded from the production of binders with alcohol ingredients (HES). The consequence will be an increase in the work comfort of the moulding plant employees and no negative impact from harmful gases resulting from the breakdown of the HES binder on the natural environment. The manufactured ceramic moulds were characterised by good physical and mechanical properties, exceeding the parameters of the reference mould.

The end result is the developed ecological technology of ceramic-casting moulds for the precise casting of aircraft engine turbine blades from nickel superalloys.

## Figures and Tables

**Table 1:** Chemical composition of ceramic mixtures for individual layers of the casting mould–HES reference system.

Mould Layer	Raw Material Composition		
	Binder	Warp	Dust
1–2	Water-based colloidal silica	Zirconium silicate + cobalt aluminate	Electrocorundum
3–7	Alcohol-hydrolysed ethyl silicate	Aluminosilicates	Aluminosilicates

**Table 2:** Summary of the average particle diameter of the tested powders.

Powder	Solvent	Average Particle Diameter [µm]
Al_2_O_3_ 200#	WATER	57.2
Al_2_O_3_ 325#	WATER	27.6
Molochite 120	WATER	51.2

## Data Availability

The original contributions presented in the study are included in the article, further inquiries can be directed to the corresponding author.

## References

[1] Rao P., Iwasa M., Tanaka T., Kondoh I. (2003). Centrifugal casting of ceramic composites of Al_2_O_3_-15% by weight ZrO_2_. Ceram. Int..

[2] Frueh C., Poirier D.R., Maguire M.C. (1997). The effect of silica-containing binders on the titanium/face coat reaction. Metall. Mater. Trans. B.

[3] Ferenc J., Michalski J., Matysiak H., Sikorski K., Kurzydlowski K.J. (2009). The influence of alumina powder on the rheological properties of zircon/silica slurries for investment casting of moulds. Proc. Inst. Mech. Eng. Part B J. Eng. Manuf..

[4] Jin W., Bai F., Li T., Yin G. (2008). Grain Refinement of Superalloy IN100 Under the Action of Rotary Magnetic Fields and Inoculants. Mater. Lett..

[5] Wang F., Zhang J., Huang T., Liu L., Fu H. (2013). Preparation of Inoculants Used in Superalloy and Analysis of the Atomic Matching Models. J. Mater. Sci. Technol..

[6] Liu L., Huang T., Xiong Y., Yang A., Zhao Z., Zhang R., Li J. (2005). Grain Refinement of Superalloy K4169 by Addition of Refiners: Cast Structure and Refinement Mechanisms. Mater. Sci. Eng. A.

[7] Du B., Yang J., Cui C., Sun X. (2015). Effects of Grain Refinement on the Microstructure and Tensile Behavior of K417G Superalloy. Mater. Sci. Eng. A.

[8] Rakoczy Ł., Cygan R. (2018). Analysis of Temperature Distribution in Shell Mould During Thin-Wall Superalloy Casting and its Effect on the Resultant Microstructure. Arch. Civ. Mech. Eng..

[9] Zupanič F., Bončina T., Križman A., Tichelaar F.D. (2001). Structure of Continuously Cast Ni-Based Superalloy Inconel 713. J. Alloys Compd..

[10] Azadi M., Marbout A., Safarloo S., Azadi M., Shariat M., Rizi M.H. (2018). Effects of Solutioning and Ageing Treatments on Properties of Inconel-713C Nickel-Based Superalloy Under Creep Loading. Mater. Sci. Eng. A..

[11] Matysiak H., Zagorska M., Balkowiec A., Adamczyk-Cieslak B., Cygan R., Cwajna J., Nawrocki J., Kurzydłowski K.J. (2014). The Microstructure Degradation of the IN 713C Nickel-Based Superalloy After the Stress Rupture Tests. J. Mater. Eng. Perform..

[12] Szczotok A., Matysiak H. (2014). Influence of Constituents of Shell Mold on the Morphology and Chemical Composition of Carbides Occurring in IN 713C Superalloy Castings. J. Mater. Eng. Perform..

[13] Szeliga D., Kubiak K., Ziaja W., Cygan R., Suchy J.S., Burbelko A., Nowak W.J., Sieniawski J. (2017). Investigation of Casting-Ceramic Shell Mold Interface Thermal Resistance During Solidification Process of Nickel Based Superalloy. Exp. Therm. Fluid Sci..

[14] Maity P., Maity J. (2001). Development of High Strength Ceramic Shell for Investment Casting. Indian Found. J..

[15] Zhang B., Sheng G., Jiao Y., Gao Z., Gong X., Fan H., Zhong J. (2017). Precipitation and Evolution of Boride in Diffusion Affected Zone of TLP Joint of MAR-M247 Superalloy. J. Alloys Compd..

[16] Małek M., Wiśniewski P., Matysiak H., Zagórska M., Kurzydłowski K.J. (2014). Technological properties of SiC-based ceramic slurries for manufacturing investment casting shell moulds. Arch. Metall. Mater..

[17] Ismael M.R., Dos Anjos R.D., Salomao R., Pandolfelli V.C. (2006). Colloidal silica as a nostructured binder for refractory castables. Refract. Appl. News.

[18] Donachie M.J., Donachie S.J. (2002). Superalloys: A Technical Guide.

[19] Heck K.A., Smith J.S., Smith R. (1998). Inconel 783: An Oxidation- Resistant, Low Expansion Superalloy for Gas Turbine Applications. J. Eng. Gas Turbines Power.

[20] Heck K.A., Smith D.F., Holderby M.A., Smith J.S. (1992). Three-Phase Controlled Expansion Superalloys with Oxidation Resistance.

[21] Furillo F.T., Davidson J., Tien J. (1979). The Effects of Grain Boundary Carbides on the Creep and Back Stress of a Nickel-Base Superalloy. Mater. Sci. Eng..

[22] Wahl F.M., Grim R.E., Graf R.B. (1961). Phase Transformations in Silica as Examined by Continuous X-ray Diffraction. Am. Mineral..

[23] Vallar S., Houivet D., El Fallah J., Kervadec D., Haussonne J.M. (1999). Oxide slurries stability and powders dispersion: Optimization with zeta potential and rheological measurements. J. Eur. Ceram. Soc..

[24] Wang Y., Guo X., Qiao Y. (2017). Interactions between Nb-Si based ultrahigh temperature alloy and yttria matrix mould shells. Mater. Des..

[25] Chen C.C.A., Vu L.T., Qiu Y.T. (2017). Study on Injection Molding of Shell Mold for Aspheric Contact Lens Fabrication. Procedia Eng..

[26] Cunha S., Tavares A., Aguiar J.B., Castro F. (2022). Cement mortars with ceramic molds shells and paraffin waxes wastes: Physical and mechanical behavior. Constr. Build. Mater..

[27] Chen S., Sun D., Wang C., Wen S., Wu J., Yan C., Shi Y., Chen C., Ren Z. (2022). Alumina-based ceramic mold with integral core and shell for hollow turbine blades fabricated by laser powder bed fusion. Addit. Manuf..

[28] (2018). Standard Test Method for Determining Specific Heat Capacity by Differential Scanning Calorimetry.

[29] Dehghanian E., Givi M.K.B., Jafari H. (2021). Recent advances in investment casting process: From conventional techniques to additive manufacturing and hybrid strategies. J. Manuf. Process..

[30] Zhang W., Huang X., Xu Y., Li M. (2023). Environmentally friendly binders for ceramic investment casting molds: A critical review. J. Eur. Ceram. Soc..

[31] Kulkarni A., Mishra S., Bandyopadhyay A. (2023). Binder formulations for sustainable ceramic processing: A review of recent developments and challenges. Ceram. Int..

